# Impact of Insulin Resistance on Insulin-Like Growth Factor-1/Insulin Like Growth Factor-Binding Protein-3 Axis and on Early Weight Gain in Small for Gestational Age Infants

**DOI:** 10.4274/Jcrpe.867

**Published:** 2013-05-30

**Authors:** Ceyhun Dizdarer, Hüseyin Anıl Korkmaz, Özlem Murat Büyükocak, Selda Mohan Tarancı, Ayşe Çoban

**Affiliations:** 1 Dr. Behçet Uz Children Disease and Surgery Training and Research Hospital, Department of Pediatric Endocrinology, İzmir, Turkey; 2 Dr. Behçet Uz Children Disease and Surgery Training and Research Hospital, Department of Pediatrics, İzmir, Turkey

**Keywords:** Well-growing, insulin-like growth factor/IGFBP-3 axis, insulin resistance, small for gestational

## Abstract

**Objective:** To assess insulin-like growth factor-1 (IGF-1)/IGF-binding protein-3 (IGFBP-3) axis and insulin resistance (IR) and the relationship of these parameters with growth in appropriate for gestational age (AGA) and small for gestational age (SGA) infants at birth and in early infancy.

M**ethods:** Postnatal blood samples for measurement of glucose, insulin, IGF-1, and IGFBP-3 were taken from 60 infants (30 AGA and 30 SGA) at birth and at one, three, and six months of age. Both SGA and AGA infants were divided into two groups: growing well and not growing well. Blood glucose, insulin, IGF-1, and IGFBP-3 values were assessed in all infants.

**Results:** Homeostasis model assessment-IR (HOMA-IR) values in well-growing SGA infants in the third and sixth months were found to be higher than in not well-growing SGA infants (3.9±0.8 vs. 1.0±0.3 at 3 months and 3.3±0.9 vs. 2.4±0.9 at 6 months, p<0.05). IGF-1 levels in well-growing SGA infants at 3 and 6 months were found to be higher than those in not well-growing SGA infants (83.80±44.50 vs. 73.50±17.60 ng/mL at 3 months and 95.12±50.74 vs. 87.67±22.91 ng/mL at 6 months, p<0.05). The IGF-1 values were significantly lower in well-growing SGA infants than in well-growing AGA infants (83.80±44.50 vs. 103.31±30.81 ng/mL at 3 months and 95.12±50.74 vs. 110.87±26.44 ng/mL at 6 months, p<0.05).

**Conclusions:** This study demonstrates the effects of accelerated early infant growth on IGF-1/IGFBP-3 axis in SGA-born infants.

**Conflict of interest:**None declared.

## INTRODUCTION

In adults who begin life as small for gestational age (SGA) infants, morbidities such as insulin resistance (IR) and type 2 diabetes mellitus, hyperlipidemia, hypertension, and coronary artery disease develop more frequently (1). In addition to unfavorable intrauterine conditions, catch-up growth in the postnatal period contributes to the development of these morbidities (2). If a rapid increase in body mass index accompanies rapid growth, especially during the first two years of life, the probability that IR will develop is high (3). IR, which is central to all morbidities listed above, develops at an early age in low-birth-weight (BW) children (4).

SGA is quite a frequent condition which is defined as restricted BW. The endocrine sequela of SGA in childhood that has received the greatest attention is short stature (5,6,7). Changes in the growth hormone (GH)/insulin-like growth factor (IGF)/IGF-binding protein (IGFBP) axis of the growth-retarded fetus and newborn have been studied extensively (6). In animal studies it has been shown that during fetal life and at birth, serum insulin and IGF-1 levels are considerably reduced and serum IGFBP-1 levels are markedly elevated in SGA models (8,9). Recent studies demonstrate that events occurring early in life can affect the GH/IGF-1 axis (10) and that impairments in the somatotropic axis contribute to a decrease in insulin sensitivity. The metabolic and hormonal changes which SGA infants develop in utero make them prone to short stature, hyperinsulinism, obesity, diabetes mellitus, metabolic syndrome, hypertension, coronary disease, exaggerated adrenarche, polycystic ovary syndrome, and many other problems.

This study was designed to demonstrate the characteristics of the IGF-1/IGFBP-3 axis and IR in appropriate for gestational age (AGA) and SGA infants by examining the metabolic and hormonal parameters in these infants. The study also aimed to demonstrate the differences in catch-up growth in SGA infants between the ages of 0 and 6 months and to compare these parameters with those in AGA infants. 

## METHODS

The study was conducted on a total of 60 AGA and SGA infants hospitalized in the neonatal and premature birth units of Izmir Dr. Behçet Uz Pediatric Disease and Surgery Training and Research Hospital. Sixty infants were enrolled in the study from January 2010 to February 2012. The cases were divided into two groups as the SGA Study Group (n=30) and the AGA Control Group (n=30). The exclusion criteria were: significant respiratory, neurological, renal, genetic, cardiovascular, hepatic, or gastrointestinal disease; systemic infections; grade 3 or 4 intraventricular hemorrhage; use of corticosteroids or diuretics within 1 week prior to enrollment; and need for mechanical ventilation or oxygen requirement of >25% (increasing FiO2 requirement because of especially cardiovascular and respiratory problems). All infants born to women with intrauterine infection, impaired glucose tolerance, gestational diabetes mellitus (GDM), chronic hypertension, obesity, multiple gestations, and smoking or alcohol consumption during pregnancy, as well as infants of mothers treated either with dextrose solutions during labor or with drugs that affect glucose metabolism, were also excluded.

All infants were followed for 6 months after birth. The infants were categorized as SGA if their BW was below the 10th percentile according to recently updated growth curves (11). SGA was defined as a BW and/or birth length below the 10th percentile for GA. AGA was defined as a BW and/or birth length between the 10th and 90th percentiles for GA (11,12). To determine the GA of infants in weeks, the modified Ballard criteria were used (12). Infants born before the 37th gestational week were considered preterm. SGA infants were divided into preterm SGA and term SGA subgroups, and AGA infants were divided into preterm AGA and term AGA subgroups. Each infant’s GA, gender, delivery method, birth order, length and weight at birth and at hospital admission, ponderal index, length and weight at their first, third and sixth months visits were recorded. In addition, the infants were divided into two subgroups: those growing well and those not growing well. “Growing well” in SGA and AGA infants was defined as a weight gain of at least 30 grams per day during months 0 to 3, and at least 20 grams per day during months 3 to 6.

The authors confirmed in writing that they have complied with the World Medical Association Declaration of Helsinki regarding ethical conduct of research involving human subjects and/or animals. The study was approved by the local ethics board and informed consent was obtained from the families of all patients.

Weight was measured using an infant scale with a precision of 10 g (Seca Model 345). Recumbent length was measured to 0.01 cm using a digital infantometer (447 Infantronic Digital Infantometer, Quickmedical). After a fasting period averaging 3 to 4 hours, 2 to 3 mL of blood were taken from all infants between the third and seventh postnatal day, the samples were immediately centrifuged to extract the serum. Blood glucose and insulin were studied immediately following blood extraction. The serum was kept at -200C until it was analyzed. IGF-1 and IGFBP-3 were analyzed from each sample. The measurements were repeated at the 1- to 3-month and 6-month follow-ups for all parameters.

Serum glucose level was measured by the hexokinase enzymatic method (Cobas Integra Kits, Roche Diagnostics, Basel, Switzerland). Insulin level was determined using the microparticle enzyme immunoassay (MEIA) (AxSYM, Abbott Laboratories, Abbott Park, IL, USA). Sensitivity level was 1.0 µU/mL. IGF-1 (ng/mL) was measured by the radioimmunoassay (RIA) method [IGF-1-RIACT (Cis bio, Gif-sur Yvette, France)] and IGFBP-3 (ng/mL) was measured by the immunoradiometric assay (IRMA) method (DSL-6600 ACTIVE, Webster, TX, USA).

We used the following indices for the determination of IR (13): the homeostasis model assessment of IR (HOMA-IR) calculated with the formula: fasting insulin (µU/mL) x fasting glucose (mmol/L)/22.5. Fasting glucose/insulin ratio (G/I) (FGIR was defined as a G/I of < 6) (14).

**Statistical Analyses**

Statistical analyses were conducted using SPSS-12.0. The chi-square test, Student’s t-test, cross-tabulation, multiple variance analyses, and Pearson covariance analysis were used. The significance level was taken as p<0.05. In cases where values could be affected by the previous measurement, the importance control of the intergroup difference was performed after refining the impact of the previous measurement by covariance analysis.

## RESULTS

The distribution of the infants is shown in Table 1. During the study, physical examination findings for all infants were normal, except for findings pertaining to their SGA state and/or prematurity.

Serum glucose levels were significantly lower in preterm SGA infants than in term SGA infants and preterm AGA infants (p<0.05), and they were significantly lower in term SGA infants than in term AGA infants (p<0.05). Blood insulin levels at birth were significantly lower in preterm SGA infants than in term SGA infants (p<0.05). However, insulin levels were significantly higher in term SGA infants than in term AGA infants (p<0.05). The HOMA-IR values were significantly lower in preterm SGA infants than in term SGA infants (p<0.05). While a significant difference was not seen in preterm SGA infants compared with preterm AGA infants (p>0.05), the HOMA-IR values were significantly higher in term SGA infants than in term AGA infants (p<0.05). However, there was no significant difference in term AGA infants when compared with preterm AGA infants (p>0.05) (Table 2). IR in SGA infants in the third and sixth months (10% and 6.6%, respectively) was higher than IR in AGA infants (3.3% and 0%, respectively) according to HOMA-IR.

When IGF-1 and IGFBP-3 values were investigated, no significant difference between preterm and term SGA infants, nor between preterm and term AGA infants (p>0.05) was detected. IGF-1 and IGFBP-3 values were significantly lower in term SGA infants than in term AGA infants (p<0.05). The same values were also significantly lower in preterm SGA infants than in preterm AGA infants (p<0.05) (Table 2). In both term and preterm infants, being SGA stood out as a factor contributing to low IGF-1 and IGFBP-3 levels.

Table 3 shows glucose, insulin, IGF-1, IGFBP-3, HOMA-IR, and FGIR values in the well-growing or not well-growing SGA and AGA infants. Insulin levels were significantly higher in well-growing SGA infants than in not well-growing infants (p<0.05). There was no significant difference between AGA and SGA infants who did not grow well (p>0.05). HOMA-IR values at 3 and 6 months were significantly higher in well-growing SGA infants compared both with not well-growing SGA-infants and with not well-growing AGA infants (p<0.05). In infants growing well and in those not growing well, no significant difference in FGIR values was detected in either study group (p>0.05) (Table 3).

When IGF-1 and IGFBP-3 levels were studied, no significant difference in growth was found in either AGA infants or SGA infants in their first month (p>0.05). Higher values were found at 3 and 6 months in well-growing SGA infants compared with those not well-growing (p<0.05) (Table 4). The IGF-1 and IGFBP-3 values were significantly lower in well-growing SGA infants than in well-growing AGA infants (p<0.05). These values were also significantly lower in SGA infants growing well than in not well-growing AGA infants (p<0.05). 

## DISCUSSION

In this prospective study, we observed a relationship between weight gain from birth to six months and glucose metabolism. Insulin values and HOMA-IR values at months 3 and 6 were significantly higher in the well-growing SGA infants compared with SGA infants who did not grow well. Interestingly, the associations to glucose metabolism were only apparent in SGA born infants, supporting the belief that growing well is adversely related with a tendency to glucose metabolism disturbances observed in these infants later in life. The study also showed that accelerated weight gain in infants born AGA did not seem to be related with disturbances of glucose metabolism. Brons et al (15) found that young men born SGA, when compared with AGA controls, had increased DNA methylation of the promoter region of the key metabolic regulator peroxisome proliferator-activated receptor gamma, coactivator 1 alpha (PPARGC1A) influencing insulin action in muscle. In addition, the men born SGA had a much lower responsiveness of DNA methylation after 5 days of overfeeding compared with AGA controls. This suggests that growth restriction early in life may alter epigenetic flexibility. However, more studies are needed to show whether DNA methylation plays any role in the programming of human metabolism.

It has been shown that GH, IGF-1, IGF-2, and IGFBP-3 levels in the second half of the gestational period in SGA fetuses were significantly lower than those in normal fetuses (16,17). In our study, we found that serum IGF-1 and IGFBP-3 values were significantly lower in SGA infants than in AGA infants at birth. This finding suggests that IGF-1 and IGFBP-3 play a major role in the control of human fetal growth (17).

In our study, we detected IGF-1 and IGFBP-3 levels to be lower in term SGA infants than in term AGA infants, in the third and sixth months of life. Our study also demonstrated that HOMA-IR in SGA infants was higher than HOMA-IR in AGA infants in the third and sixth months. We thought that in well-growing SGA infants, IR leads to compensatory hyperinsulinemia which in turn increases circulating IGF-1 and possibly IGFBP-3 levels. In our study, higher IGF-1 and IGFBP-3 values were found at 3 and 6 months in well-growing SGA infants compared with not well-growing infants. The positive association between fasting insulin and fasting IGF-1 levels in our study supports this hypothesis. Insulin has important regulatory effects on the GH/IGF-1 axis (18,19,20). Insulin is thought to regulate circulating IGF-1 levels by facilitating GH binding to the GH receptor in the liver (21). Furthermore, insulin stimulates IGF-1 mRNA production in cultured hepatocytes, possibly by exerting its effect at the transcriptional level (22). Insulin may increase circulating IGFBP-3 levels by reducing IGFBP-3 degradation (23). We suggest that there is also an IR to IGFBP-1 regulation in SGA infants and that hyperinsulinism secondary to IR may have led to these changes in the IGF-IGFBP axis in SGA infants.

Evaluation of HOMA-IR in our study demonstrated that IR in well-growing SGA infants in the third and sixth months was higher than in not well-growing SGA infants. We speculate that well-growing SGA infants in their early infancy have lower growth tendencies in later life because of changes in IGF-IGFBP axis and thus are prone to have shorter adult heights; ensuring maximal growth would directly influence their future quality of life. The relationship between the etiology of shorter adult height of SGA infants and IGF-1 and IGFBP-3 makes the use of GH a current issue in the treatment of short stature in these infants. Treatment of SGA infants with GH for GH deficiency and for short stature may be important, both to ensure their optimal growth and to minimize the effect of IGF-1 and IGFBP-3 deficiency on IR.

Detection of significantly higher IGF-1 and IGFBP-3 levels in SGA infants with catch-up growth and in well-growing AGA infants demonstrates that the factors in question affect not only intrauterine but postnatal life as well. Soto et al. detected higher IGF-1 levels in SGA infants with catch-up growth than in SGA infants without catch-up growth (4). In our study, both IGF-1 and IGFBP-3 levels at the third and sixth months in infants with catch-up growth were also found to be significantly higher in both SGA and AGA infants. Since IR accompanies this growth, it is important that weight gain be carefully monitored in well-growing infants with catch-up growth, especially in SGA infants, and that these infants be protected from this disadvantage.

One weak point of this study is that the numbers in some subgroups are quite low.

In conclusion, we believe that this study has shown the effects of accelerated early infant growth on the IGF/IGFBP-3 axis in SGA-born infants. Our findings include increased basal insulin levels associated with weight gain between birth and six months of age, and a positive association between insulin secretion and growth status at one, three and six months of age. Well-growing SGA infants are adversely affected and have a tendency for disturbances of glucose metabolism later in life.

**Acknowledgements**

The study was supported by the Dr.Behcet Uz Children Disease and Surgery Training and Research Hospital financial committee. We thank Dr. Samim Özen for his support.

## Figures and Tables

**Table 1:**
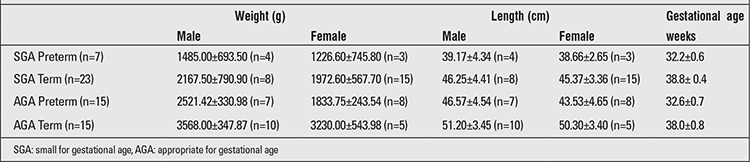
Characteristics of AGA and SGA infants at the birth

**Table 2:**
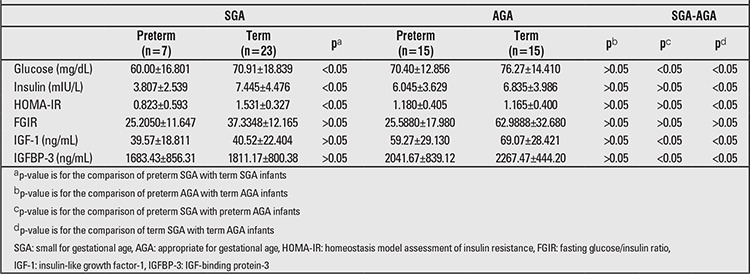
Biochemical measurements in SGA and AGA infants at the birth

**Table 3:**
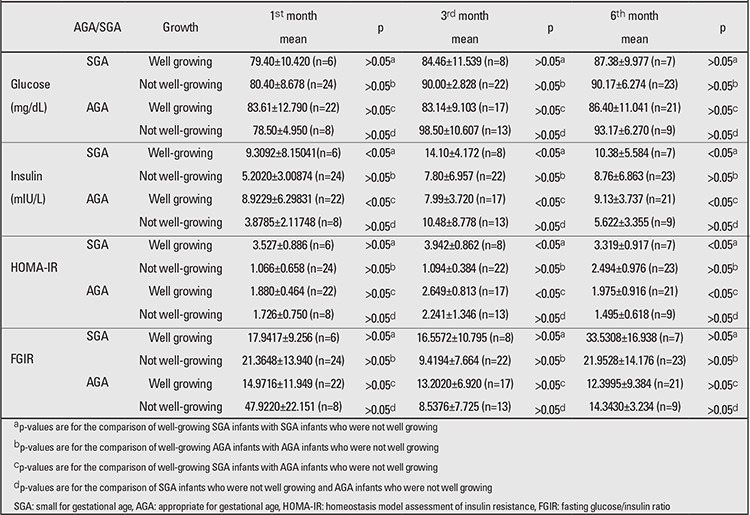
Biochemical measurements in AGA and SGA infants

**Table 4:**
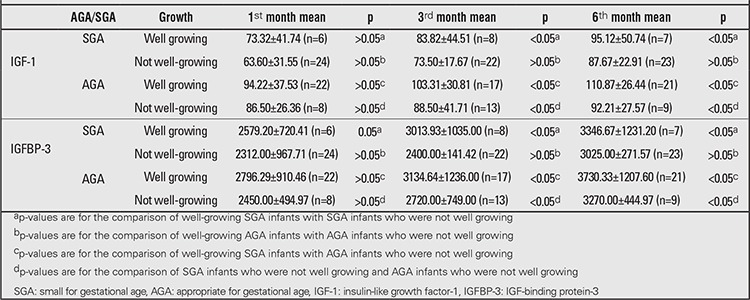
IGF-1 and IGFBP-3 measurements in AGA and SGA infants
